# Defining Factors Associated with High-quality Surgery Following Radical Cystectomy: Analysis of the British Association of Urological Surgeons Cystectomy Audit

**DOI:** 10.1016/j.euros.2021.08.005

**Published:** 2021-09-20

**Authors:** Wei Shen Tan, Jeffrey J. Leow, Maya Marchese, Ashwin Sridhar, Giles Hellawell, Matthew Mossanen, Jeremy Y.C. Teoh, Sarah Fowler, Alexandra J. Colquhoun, Jo Cresswell, James W.F. Catto, Quoc-Dien Trinh, John D. Kelly, on behalf of the BAUS Section of Oncology

**Affiliations:** a Division of Surgery & Interventional Science, University College London, London, UK; b Department of Urology, Royal Free London NHS Foundation Trust, London, UK; c Department of Urology, University College London Hospitals NHS Foundation Trust, London, UK; d Department of Urology, Tan Tock Seng Hospital, Singapore; e Lee Kong Chian School of Medicine, Nanyang Technological University, Singapore; f Center for Surgery and Public Health, Division of Urological Surgery, Brigham and Women’s Hospital, Harvard Medical School, Boston, MA, USA; g Department of Urology, Northwick Park Hospital, London North West University Healthcare NHS Trust, London, UK; h Lank Center for Genitourinary Oncology, Dana-Farber/Brigham and Women's Cancer Center, Harvard Medical School, Boston, MA, USA; i The S H Ho Urology Centre, Department of Surgery, The Chinese University of Hong Kong, Hong Kong; j British Association of Urological Surgeons, London, UK; k Department of Urology, Addenbrooke's Hospital, Cambridge University Hospitals NHS Foundation Trust, Cambridge, UK; l Department of Urology, James Cook University Hospital, South Tees Hospitals NHS Foundation Trust, Middlesbrough, UK; m Academic Urology Unit, University of Sheffield, Sheffield, UK

**Keywords:** British Association of Urological Surgeons audit, Bladder cancer, Centralisation, Quality surgery, Radical cystectomy, Outcomes

## Abstract

**Background:**

Radical cystectomy (RC) is associated with high morbidity.

**Objective:**

To evaluate healthcare and surgical factors associated with high-quality RC surgery.

**Design, setting, and participants:**

Patients within the prospective British Association of Urological Surgeons (BAUS) registry between 2014 and 2017 were included in this study.

**Outcome measurements and statistical analysis:**

High-quality surgery was defined using pathological (absence of positive surgical margins and a minimum of a level I lymph node dissection template with a minimum yield of ten or more lymph nodes), recovery (length of stay ≤10 d), and technical (intraoperative blood loss <500 ml for open and <300 ml for minimally invasive RC) variables. A multilevel hierarchical mixed-effect logistic regression model was utilised to determine the factors associated with the receipt of high-quality surgery and index admission mortality.

**Results and limitations:**

A total of 4654 patients with a median age of 70.0 yr underwent RC by 152 surgeons at 78 UK hospitals. The median surgeon and hospital operating volumes were 23.0 and 47.0 cases, respectively. A total of 914 patients (19.6%) received high-quality surgery. The minimum annual surgeon volume and hospital volume of ≥20 RCs/surgeon/yr and ≥68 RCs/hospital/yr, respectively, were the thresholds determined to achieve better rates of high-quality RC. The mixed-effect logistic regression model found that recent surgery (odds ratio [OR]: 1.22, 95% confidence interval [CI]: 1.11–1.34, *p* < 0.001), laparoscopic/robotic RC (OR: 1.85, 95% CI: 1.45–2.37, *p* < 0.001), and higher annual surgeon operating volume (23.1–33.0 cases [OR: 1.54, 95% CI: 1.16–2.05, *p* = 0.003]; ≥33.1 cases [OR: 1.64, 95% CI: 1.18–2.29, *p* = 0.003]) were independently associated with high-quality surgery. High-quality surgery was an independent predictor of lower index admission mortality (OR: 0.38, 95% CI: 0.16–0.87, *p* = 0.021).

**Conclusions:**

We report that annual surgeon operating volume and use of minimally invasive RC were predictors of high-quality surgery. Patients receiving high-quality surgery were independently associated with lower index admission mortality. Our results support the role of centralisation of complex oncology and implementation of a quality assurance programme to improve the delivery of care.

**Patient summary:**

In this registry study of patients treated with surgical excision of the urinary bladder for bladder cancer, we report that patients treated by a surgeon with a higher annual operative volume and a minimally invasive approach were associated with the receipt of high-quality surgery. Patients treated with high-quality surgery were more likely to be discharged alive following surgery.

## Take Home Message

In this UK registry study of bladder cancer patients treated with radical cystectomy, we report that patients treated by a surgeon with a higher annual operative volume and a minimally invasive approach were associated with the receipt of high-quality surgery. Patients treated with high-quality surgery were more likely to be discharged alive following surgery.

## 1 Introduction

Radical cystectomy (RC) with lymph node dissection (LND) is the recommended treatment for muscle-invasive bladder cancer (MIBC) and selected high-risk non–muscle-invasive bladder cancer [1], [2]. RC can be a technically challenging procedure, often performed in patients with pre-existing comorbidities [3] with a competing risk of mortality [4]. Complications and morbidity after RC are common [5], and a variety of factors influence postoperative oncological and nononcological outcomes [6], [7].

Efforts to improve outcomes from RC through standardised care pathways have been included in various guidelines [8], [9] and have led to the centralisation of complex pelvic cancer services in the UK [10], [11], [12]. Despite these approaches, outcomes following RC vary widely [11], [13], reflecting differences in practice, case volume-outcome relationships, and adherence to guidelines. Nevertheless, variation of care following RC despite centralisation of care remains, suggesting that a magnitude of factors, other than the volume-outcome relationship, influence perioperative outcomes.

Defining high-quality surgery is a subject of debate, but there are several uniform consensuses that we have identified in our recent collaborative review [14]. While cancer stage and positive lymph node status influence cancer survival rates, surgical technique to minimise positive surgical margin (PSM) status and adequate LND remains crucial [15]. Hence, the absence of a pathological PSM [14], [16] and a template LND [14], [17] with a minimum LND count is paramount [15]. A surrogate for surgical technique would be surgical blood loss [18] and hospital length of stay (LOS) [19], which would be impacted by perioperative complications [20]. LOS was used as a surrogate of enhanced recovery pathways and clinically meaningful complications, and can be used as a surrogate measure of recovery [21], [22], [23], [24].

In 2013, the British Association of Urological Surgeons (BAUS) mandated that all RCs performed in the UK should be recorded prospectively in a registry. We utilised this database to interrogate factors associated with our predefined high-quality surgery indicators.

## 2 Patients and methods

### 2.1 Patient selection

Patients included in this analysis were nonmetastatic bladder cancer patients who underwent RC between January 2014 and December 2017 across the UK and entered into the BAUS registry. Data were prospectively self-submitted by individual surgeons (or their hospital delegates). Surgeons were provided two opportunities to check/validate the data recorded to ensure accuracy. Cases within the registry represent approximately >80% of all RC surgeries performed in the UK according to NHS data [25].

### 2.2 Variables of interest

The following patient-specific variables were extracted: patient age at diagnosis (continuous), gender (male and female), Charlson Comorbidity Index (CCI; 0, 1, 2, and ≥3), anaerobic threshold (AT) based on cardiopulmonary exercise tolerance testing (<11 and ≥11), body mass index (BMI) by quartiles (≤24.7, 24.8–27.4, 27.5–30.5, and ≥30.6), preoperative anaemia (yes or no), preoperative chemotherapy use (yes or no), preoperative radiotherapy use (yes or no), hospital LOS, and year of surgery (2014, 2015, 2016, and 2017).

The surgical-related factors extracted included the following: urinary diversion type (ileal conduit, continent diversion, and other), surgical approach (open and minimally invasive [robotic/laparoscopic]), operating surgeon volume by quartiles (≤14, 14.1–23.0, 23.1–33.0, and ≥33.1), hospital case volume by quartiles (≤29.0, 29.1–47.0, 47.1–63.0, and ≥63.1), estimated blood loss (≤299, 300–499, 500–999, and ≥1000 ml), requirement for blood transfusion (yes or no), and number of pack red blood cells transfused (1–2 and ≥3 units). Cancer-specific variables extracted included the following: cancer grade (G1/2 and G3), clinical cancer stage (≤cT1, cT2, and cT3/4), clinical nodal stage (cN1+ and cN0), PSM status (yes or no), and adequate LND (yes or no).

**Fig. 1:**
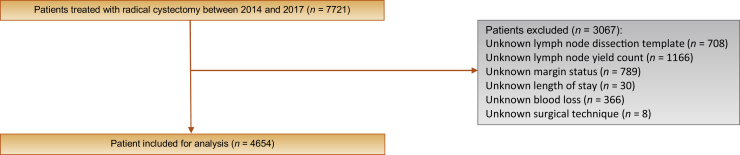
Inclusion and exclusion criteria used to determine study cohort.

Patients with missing data for the following variables were excluded from analysis: LND template, lymph node yield count, PSM status, hospital LOS, surgical technique, blood loss, and inpatient mortality (Fig. 1).

### 2.3 High-quality surgery

High-quality surgery was defined as surgery in patients who had (1) a negative surgical margin, (2) adequate LND, (3) hospital LOS ≤ 10 d, and (4) minimal intraoperative estimated blood loss [15], [26]. PSM was defined as any soft tissue or carcinoma in situ (CIS) at surgical margin. Adequate LND was defined as a minimum of level I LND with a lymph node yield of ten or more nodes. Minimal blood loss was defined as <500 ml blood loss for open RC cases and <300 ml for minimally invasive RC cases.

The variables that we identified to determine high-quality surgery were defined based on well-validated variables of good outcomes following surgery. PSM has been shown to be independently associated with cancer-specific survival following RC [14], [16]. We defined the removal of a minimum of ten or more lymph nodes at RC as a benchmark, as it has been shown to be a predictor of a superior oncological outcome [15]. This is in combination with a minimum of a level I LND as an extended LND is not superior to a limited LND [14], [17]. A hospital LOS threshold of ≤10 d was used, as it represents the median LOS of the cohort. Intraoperative blood loss is often a surrogate for high-quality surgery and may be associated with increased mortality in noncardiac surgery [19]. The threshold of ≤300 ml was suggested in the Pasadena Consensus to define experienced robot-assisted RC (RARC) surgeons [18], [27]. RARC has been shown to be associated with lower blood loss than open RC; hence, we accounted for this using different thresholds when defining quality surgery [28].

### 2.4 Statistical analysis

Categorical variables were reported using descriptive statistics, frequency, and proportions. Continuous variables were reported using median and interquartile range (IQR). To determine bivariate differences between patient groups, χ^2^ and Wilcoxon tests were used for categorical and continuous variables, respectively. Annual hospital volume and surgeon volume were categorised to deciles, and minimally required annual surgical volume to achieve higher high-quality surgery was determined using the minimum *p*-value approach [29]. To adjust for clustering within treating hospitals, we utilised a random-effect model to account for individual treating hospitals [30], [31]. Subsequently, a mixed-effect logistic regression model was utilised to predict the odds of a patient receiving high-quality surgery and index admission mortality following adjustment for patient-, cancer-, and hospital-related factors. Sensitivity analysis was performed to determine the association between hospital LOS and index admission mortality to ensure shorter hospital LOS, which was a quality matrix and was not attributed to early postoperative mortality. Hospitals were then ranked from least likely to most likely achieving high-quality surgery following RC and plotted against the probability of high-quality surgery to obtain a caterpillar graph.

Data analysis was performed using Stata 15 (StataCorp, College Station, TX, USA). A two-sided *p* value of <0.05 was considered statistically significant. This project was approved by the BAUS Oncology Council. In accordance with the UK National Research Ethics Service guidelines, ethical approval was not required.

## 3 Results

### 3.1 Cohort

A total of 4654 patients with a median age of 70.0 (IQR: 63.0–75.0) yr underwent RC at 78 institutions by 152 surgeons. The median annual RC caseload was 23.0 (IQR: 14.0–33.0) cases per surgeon and 47.0 (IQR: 29.0–63.0) cases per hospital. A total of 1731 patients (37.2%) at 42 hospitals received minimally invasive RC. Of the 2775 MIBC patients, a total of 667 (24.0%) received neoadjuvant chemotherapy (NAC), while 268 (5.8%) had a salvage RC after radiotherapy. Continent diversion was constructed for 293 patients (6.3%).

### 3.2 Outcomes in quality metrics

A total of 455 patients (12.2%) had a PSM, including 222 patients (5.9%) with soft tissue/circumferential involvement and 119 (3.2%), 83 (2.2%), and 31 (0.8%) patients with ureteric, urethral, and unknown margins, respectively (Table 1). The soft tissue PSM rate for pT2 patients was 0.5%. The median lymph node yield was 13.0 (IQR: 8.0–19.0), and 3214 patients (69.1%) had a lymph node yield of ten or more nodes. A total of 3142 patients (67.5%) met our “adequate LND” definition. Mortality following admission for RC was observed in 1.8%, and the median hospital LOS was 10.0 (IQR: 7.0–14) d. A total of 914 patients (19.6%) who were operated by 109 surgeons (71.7%) at 65 hospitals (83.3%) fulfilled all four *high-quality surgery* metrics.

**Table 1:** Baseline patient, hospital, and cancer-specific variables stratified by high-quality surgery status

	All patients (*n* = 4654)	High-quality surgery (*n* = 914)	Not high-quality surgery (*n* = 3740)	*p* value
Age at diagnosis (yr), mean ± standard error mean	69.7 ± 0.3	68.1 ± 0.6	70.1 ± 0.3	0.002
Gender, *n* (%)				0.195
Male	3495 (75.1)	702 (76.8)	2793 (74.7)	
Female	1152 (24.8)	212 (23.2)	940 (25.1)	
Unknown	7 (0.1)	0 (0)	7 (0.2)	
Charlson Comorbidity Index, *n* (%)				<0.001
0	1924 (41.4)	417 (45.6)	1507 (40.3)	
1	687 (14.8)	134 (14.7)	553 (14.8)	
2	756 (16.2)	135 (14.8)	621 (16.6)	
≥3	620 (13.3)	85 (9.3)	535 (14.3)	
Unknown	667 (14.3)	143 (15.6)	524 (14.0)	
Anaerobic threshold, *n* (%)				0.006
<11	380 (8.2)	73 (8.0)	307 (8.2)	
≥11.1	628 (13.5)	153 (16.7)	475 (12.7)	
Unknown	3646 (78.3)	688 (75.3)	2958 (79.1)	
BMI, *n* (%)				<0.001
≤24.7	848 (18.2)	187 (20.5)	661 (17.7)	
24.8–27.4	879 (18.9)	187 (20.5)	692 (18.5)	
27.5–30.5	861 (18.5)	174 (19.0)	687 (18.4)	
≥30.6	851 (18.3)	121 (13.2)	730 (19.5)	
Unknown	1215 (26.1)	245 (26.8)	970 (25.9)	
Neoadjuvant chemotherapy use, *n* (%)				<0.001
No	1100 (23.6)	261 (28.5)	839 (22.4)	
Yes	667 (14.4)	155 (17.0)	512 (13.7)	
Unknown	2887 (62.0)	498 (54.5)	2389 (63.9)	
Preoperative radiotherapy, *n* (%)				<0.001
No	3672 (78.9)	794 (86.9)	2878 (76.9)	
Yes	268 (5.8)	18 (2.0)	250 (6.7)	
Unknown	714 (15.3)	102 (11.1)	612 (16.4)	
Year of surgery, *n* (%)				<0.001
2014	1037 (22.3)	136 (14.9)	901 (24.1)	
2015	1216 (26.1)	260 (28.4)	956 (25.5)	
2016	1247 (26.8)	246 (26.9)	1001 (26.8)	
2017	1154 (24.8)	272 (29.8)	882 (23.6)	
Urinary diversion type, *n* (%)				<0.001
Ileal conduit	4217 (90.6)	867 (94.9)	3350 (89.6)	
Continent	293 (6.3)	32 (3.5)	261 (7.0)	
Other	89 (1.9)	5 (0.5)	84 (2.2)	
Unknown	55 (1.2)	10 (1.1)	45 (1.2)	
Surgical approach, *n* (%)				<0.001
Open	2923 (62.8)	413 (45.2)	2510 (67.1)	
Laparoscopic/robotic	1731 (37.2)	501 (54.8)	1230 (32.9)	
Annual surgeon operating volume, *n* (%)				<0.001
≤14.0	1196 (25.7)	167 (18.3)	1029 (27.5)	
14.1–23.0	1275 (27.4)	233 (25.5)	1042 (27.8)	
23.1–33.0	1036 (22.3)	248 (27.1)	788 (21.1)	
≥33.1	1147 (24.6)	266 (29.1)	881 (23.6)	
Annual hospital operating volume, *n* (%)				0.001
≤29.0	1268 (27.3)	202 (22.1)	1006 (28.5)	
29.1–47.0	1132 (24.3)	241 (26.4)	891 (23.8)	
47.1–63.0	1201 (25.8)	241 (26.4)	960 (25.7)	
≥63.1	1053 (22.6)	230 (25.1)	823 (22.0)	
Blood loss (ml), *n* (%)				<0.001
≤299	1444 (31.0)	646 (70.7)	798 (21.3)	
300–499	1361 (29.2)	268 (29.3)	1093 (29.2)	
500–999	1222 (26.3)	0 (0)	1222 (32.7)	
≥1000	627 (13.5)	0 (0)	627 (16.8)	
Red blood cell transfusion, *n* (%)				<0.001
No	3838 (82.5)	875 (95.7)	2963 (79.2)	
Yes	742 (15.9)	30 (3.3)	712 (19.1)	
Unknown	74 (1.6)	9 (1.0)	65 (1.7)	
Red blood cell transfusion, *n* (%)				<0.001
No	3838 (82.5)	875 (95.7)	2963 (79.2)	
1–2 units	525 (11.3)	29 (3.2)	496 (13.3)	
≥3 units	217 (4.6)	1 (0.1)	216 (5.8)	
Unknown	74 (1.6)	9 (1.0)	65 (1.7)	
Tumour grade, *n* (%)				0.733
Low	306 (6.6)	55 (6.0)	251 (6.7)	
High	3578 (76.9)	709 (77.6)	2869 (76.7)	
Unknown	770 (16.5)	150 (16.4)	620 (16.6)	
Pathological T stage, *n* (%)				0.043
≤pT1	1889 (40.6)	390 (42.7)	1499 (40.0)	
pT2	786 (16.9)	169 (18.5)	617 (16.5)	
pT3–4	1833 (39.4)	323 (35.3)	1510 (40.4)	
Unknown	146 (3.1)	32 (3.5)	114 (3.0)	
Pathological N stage, *n* (%)				<0.001
pN0	3411 (73.3)	729 (79.7)	2682 (71.7)	
pN+	916 (19.7)	166 (18.2)	750 (20.1)	
Unknown	327 (7.0)	19 (2.1)	308 (8.2)	
Type of positive surgical margin, *n* (%)				
Vesical tissue	222 (5.9)		222 (5.9)	
Ureteric	119 (3.2)		119 (3.2)	
Urethral	83 (2.2)		83 (2.2)	
Unknown	31 (0.8)		31 (0.8)	

BMI = body mass index.

### 3.3 Factors associated with high-quality surgery

Table 1 reports baseline patient, hospital, and cancer-specific variables stratified by high-quality surgery status. Patients who were younger (*p* = 0.035); had a lower CCI (*p* < 0.001), higher AT (*p* = 0.006), and lower BMI (*p* < 0.001); did not receive NAC (*p* < 0.001); did not receive radiotherapy (*p* < 0.001); had more recent surgery (*p* < 0.001); received ileal conduit (*p* < 0.001); were operated by a minimally invasive approach (*p* < 0.001); had higher annual surgeon (*p* < 0.001) and hospital (*p* = 0.001) operating volume; had minimal blood loss (*p* < 0.001); did not require red blood cell transfusion (*p* < 0.001); had a lower number of transfused blood units (*p* < 0.001); and were with absence of lymph node disease (*p* = 0.025) were significantly associated with the attainment of high-quality surgery. Minimum annual surgeon and hospital volumes of ≥20 cystectomies/surgeon/yr and ≥68 cystectomies/hospital/yr were, respectively, the thresholds determined to achieve better rates of high-quality RC (Supplementary Table 1).

**Fig. 2:**
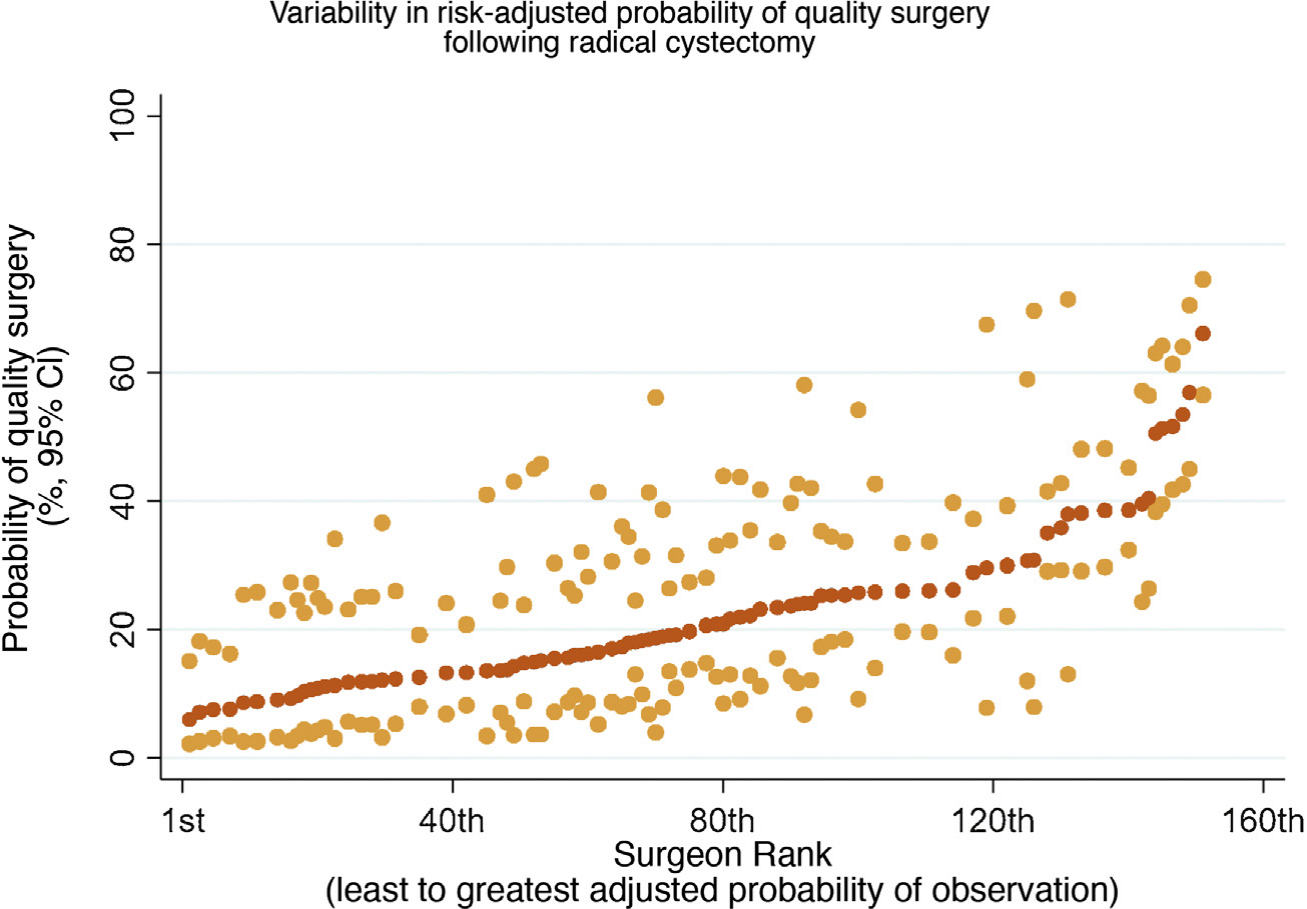
Caterpillar graph of adjusted probability of individual surgeons achieving high-quality surgery ranked from the least to the greatest. CI = confidence interval.

Multilevel hierarchical mixed-effect logistic regression was utilised to determine variables independently associated with high-quality surgery (Table 2). Older patients (odds ratio [OR]: 0.99, 95% confidence interval [CI]: 0.99–1.00, *p* = 0.010), higher CCI (≥3 [OR: 0.64, 95% CI: 0.47–0.85, *p* = 0.005]), higher BMI (≥30.6 [OR: 0.50, 95% CI: 0.38–0.67]), pN+ (OR: 0.77, 95% CI: 0.62–0.97, *p* = 0.023), preoperative radiotherapy (OR: 0.35, 95% CI: 0.21–0.58, *p* < 0.001), and continent diversion (OR: 0.38, 95% CI: 0.25–0.57, *p* < 0.001) were independently associated with a lower likelihood of high-quality surgery. Minimally invasive (laparoscopic/robotic) RC (OR: 1.85, 95% CI: 1.45–2.37, *p* < 0.001), higher annual surgeon operating volume (23.1–33.0 [OR: 1.54, 95% CI: 1.16–2.05, *p* = 0.003]; ≥33.1 [OR: 1.64, 95% CI: 1.18–2.29, *p* = 0.003]), and more recent surgery year (OR: 1.22, 95% CI: 1.11–1.34, *p* < 0.001) were independently associated with receiving high-quality surgery. The sensitivity analysis performed following the exclusion of AT, BMI, and NAC from the multilevel hierarchical mixed-effect logistic regression model due to >20% missing values reaffirms our findings. Figure 2 shows a caterpillar plot depicting the individual surgeon-adjusted risk of high-quality surgery following adjustment for other factors.

**Table 2:** Multilevel hierarchical mixed-effect logistic regression model to determine variables independently associated with high-quality surgery in bladder cancer patients treated with radical cystectomy

Variables	Odds ratio	95% CI	*p* value
*Patient-specific variables*			
Age (continuous)	0.91	0.012	0.84–0.98
Charlson Comorbidity Index			
0	Reference	Reference	
1	0.85	0.67–1.08	0.190
2	0.80	0.62–1.03	0.083
≥3	0.65	0.48–0.88	0.005
Unknown	0.81	0.58–1.13	0.219
Anaerobic threshold			
<11	Reference	Reference	
≥11.1	1.36	0.95–1.95	0.090
Unknown	1.43	0.53–1.17	0.042
BMI			
≤24.7	Reference	Reference	
24.8–27.4	0.81	0.63–1.05	0.111
27.5–30.5	0.78	0.60–1.01	0.057
≥30.6	0.50	0.38–0.67	<0.001
Unknown	0.89	0.67–1.17	0.408
Neoadjuvant chemotherapy use			
No	Reference	Reference	
Yes	0.93	0.69–1.24	0.599
Unknown	0.69	0.57–0.85	<0.001
Preoperative radiotherapy			
No	Reference	Reference	
Yes	0.35	0.21–0.59	<0.001
Unknown	0.88	0.65–1.20	0.416
Urinary diversion type			
Ileal conduit	Reference	Reference	
Continent	0.37	0.25–0.57	<0.001
Other	0.35	0.14–0.92	0.033
Unknown	0.89	0.41–1.90	0.756
Year of surgery (continuous)	1.22	1.11–1.34	<0.001
*Cancer-specific variables*			
Tumour grade			
Low	Reference	Reference	
High	0.97	0.69–1.36	0.843
Unknown	0.79	0.53–1.17	0.234
Pathological T stage			
≤pT1	Reference	Reference	
pT2	1.01	0.80–1.27	0.945
pT3–4	0.85	0.69–1.04	0.114
Unknown	1.85	1.12–3.05	0.016
Pathological N stage			
pN0	Reference	Reference	
pN+	0.77	0.62–0.97	0.023
Unknown	0.26	0.15–0.43	<0.001
*Hospital-level variables*			
Surgical approach			
Open	Reference	Reference	
Laparoscopic/robotic	1.86	1.46–2.38	<0.001
Annual surgeon operating volume			
≤14	Reference	Reference	
14.1–23.0	1.30	0.99–1.70	0.060
23.1–33.0	1.54	1.16–2.04	0.003
≥33.1	1.64	1.18–2.28	0.003
Annual hospital operating volume			
≤29.0	Reference	Reference	
29.1–47.0	0.94	0.68–1.29	0.685
47.1–63.0	0.80	0.55–1.16	0.238
≥63.1	0.75	0.43–1.28	0.290

BMI = body mass index; CI = confidence interval.

We subsequently confirmed, using multilevel hierarchical mixed-effect logistic regression, that patients who achieved high-quality surgery were significantly less likely to have index admission mortality (OR: 0.38, 95% CI: 0.16–0.87, *p* = 0.021; Table 3). This finding was consistent even after the exclusion of AT, BMI, and NAC from the multilevel hierarchical mixed-effect logistic regression model (OR: 0.37, 95% CI: 0.16–0.85, *p* = 0.019). A sensitivity analysis suggests that the mean hospital LOS was significantly shorter in patients discharged alive than in patients who had an inpatient death (12.9 vs 28.2 d, *p* < 0.001). Other factors associated with an increased risk of index admission death include pT3–4 (OR: 2.17, 95% CI: 1.20–3.94, *p* = 0.011) and ≥3 units of blood transfused (OR: 3.5, 95% CI: 1.54–8.17, *p* = 0.003).

**Table 3:** Multilevel hierarchical mixed-effect logistic regression model to determine variables independently associated with inpatient mortality in bladder cancer patients treated with radical cystectomy

Variables	Odds ratio	95% CI	*p* value
High-quality surgery	0.38	0.16–0.87	0.021
*Patient-specific variables*			
Age (continuous)	1.02	1.00–1.04	0.024
Charlson Comorbidity Index			
0	Reference	Reference	
1	1.05	0.53–2.09	0.894
2	1.09	0.55–2.16	0.807
≥3	1.92	1.00–3.70	0.051
Unknown	0.74	0.30–1.81	0.503
Anaerobic threshold			
<11	Reference	Reference	
≥11.1	1.12	0.46–2.70	0.805
Unknown	0.74	0.34–1.62	0.452
Body mass index			
≤24.7	Reference	Reference	
24.8–27.4	0.62	0.30–1.27	0.191
27.5–30.5	0.57	0.27–1.21	0.145
≥30.6	0.61	0.29–1.28	0.192
Unknown	0.97	0.50–1.88	0.938
Neoadjuvant chemotherapy use			
No	Reference	Reference	
Yes	0.74	0.31–1.78	0.496
Unknown	1.14	0.65–2.01	0.642
Preoperative radiotherapy			
No	Reference	Reference	
Yes	0.82	0.33–2.03	0.662
Unknown	1.34	0.61–2.92	0.464
Year of surgery (continuous)	1.13	0.85–1.51	0.393
Blood loss (ml)			
≤299	Reference	Reference	
300–499	1.03	0.56–1.87	0.930
500–999	0.55	0.26–1.14	0.107
≥1000	0.55	0.22–1.33	0.183
Red blood cell transfusion			
No	Reference	Reference	
1–2 units	1.72	0.87–3.39	0.118
≥3 units	3.54	1.54–8.17	0.003
Unknown	2.95	0.77–11.24	0.113
*Cancer-specific variables*			
Tumour grade			
Low	Reference	Reference	
High	1.71	0.52–5.61	0.374
Unknown	2.22	0.61–8.06	0.227
Pathological T stage			
≤pT1	Reference	Reference	
pT2	1.78	0.87–3.68	0.117
pT3–4	2.17	1.20–3.94	0.011
Unknown	2.19	0.65–7.37	0.205
Pathological N stage			
pN0	Reference	Reference	
pN+	1.03	0.59–1.82	0.911
Unknown	1.49	0.67–3.31	0.323
*Hospital-level variables*			
Surgical approach			
Open	Reference	Reference	
Laparoscopic/robotic	1.13	0.62–2.08	0.691
Annual surgeon operating volume			
≤14	Reference	Reference	
14.1–23.0	0.82	0.44–1.53	0.534
23.1–33.0	0.96	0.47–1.96	0.913
≥33.1	0.75	0.31–1.82	0.524
Annual hospital operating volume			
≤29.0	Reference	Reference	
29.1–47.0	0.97	0.49–1.95	0.937
47.1–63.0	1.09	0.53–2.27	0.809
≥63.1	0.82	0.32–2.10	0.679

CI = confidence interval.

## 4 Discussion

We report that one in five patients who underwent RC between 2014 and 2017 achieved our definition of high-quality surgery. Annual surgeon operating volume (but not annual hospital operating volume) was a predictor of the attainment of high-quality surgery. Minimally invasive RC and more recent year of surgery were other factors that predicted achievement of high-quality surgery. Patients who received high-quality surgery were more likely to be discharged alive following RC. Significant variability exists at an individual surgeon level in the attainment of high-quality surgery.

Defining high-quality surgery is essential to prove good-quality care. Particularly in the case for RC, a complex procedure with high morbidity and mortality, identification of providers of high-quality surgery care would allow clinicians and administrators to audit and improve performance. Acknowledging limitations of retrospective data, this study is important to promote organisation of complex cancer surgery and may be useful in the selection of centres of excellence, based on the hub-and-spoke model. In healthcare models that are based on fee for service models, such high-quality matrix may help inform patient’s choice of hospital as well as guide insurance companies in deciding on preferred centres for referral.

Efforts to improve cancer outcomes have led to the centralisation of pelvic oncology services in the UK that commenced in 2002, where centres had to perform a minimum of 50 pelvic oncology cases per annum [32]. Between 2003 and 2013, the number of hospitals performing RC has decreased steadily with a corresponding increase in the number of cases performed per hospital, with >95% of cases being compliant with National Institute for Health and Care Excellence (NICE) recommendations [33]. The development of high-volume surgeons and hospitals has subsequently resulted in a reduction of 90-d mortality rates from 5.8% to 2.6% [33]. Indeed, our results suggest a strong correlation between annual surgeon operating volumes independent of confounding factors, although we did not observe this for annual hospital surgical volume. This suggests that intersurgeon variation whether in a form of operative technique or in terms of the postoperative convalescent period may be crucial. This highlights the importance of the implementation of a quality assurance programme to improve the collective outcome of patient case [34].

In a collaborative review that we recently published, we recommended that surgical quality indicators should include selection for continent diversion, receipt of NAC, adequacy of LND, blood loss, operative time, negative surgical margins, and standardised morbidity and mortality reporting [14]. Other groups have reported quality assessment tools to evaluate the performance of RC. Hussein et al [35] utilised variables including the use of NAC, operative time (<6.5 h) and blood loss (<500 ml), negative soft tissue surgical margins and lymph node yield (≥20), and freedom from high-grade complications, readmission, and noncancer 30-d mortality. Their quality cystectomy score was independently associated with cancer-specific, recurrence-free, and overall survival in robotic cystectomy patients [35]. Khanna et al [36] utilised the National Cancer Database to define a bladder cancer quality score (BCQS) utilising the following variables: PSM, LND, unplanned readmission rate ≤30 d from discharge, proportion of MIBC patients receiving NAC, proportion of patients receiving continent urinary diversion, postoperative LOS, and time of diagnosis from cystectomy. They reported that academic institutions were associated with a better BCQS, and this in turn was associated with lower 90-d disease-specific (HR: 0.84, 95% CI: 0.72–0.97) and overall (HR: 0.86, 95% CI: 0.81–0.92) mortality. However, what constitutes a good BCQS was not defined. Similarly, our high-quality surgery matrix was an independent predictor of lower index admission mortality.

We acknowledged limitations to our study. The data submitted to the BAUS cystectomy audit are normally self-submitted by the surgeon or administrative staff at the institution, which may lead to a reporting bias. While it is mandated by the BAUS, up to 20% of cases may not have been recorded, and this may mean that the reported outcomes might not reflect lower-volume surgeons that may have potentially worse outcomes. We acknowledge that the dataset does not capture accurately the use of enhanced recovery after surgery, 30- and 90-d mortality, and complication rate, as well as time from transurethral resection of bladder tumour to RC. Hence, we could not correlate high-quality surgery matrix with survival outcomes and utilised a surrogate of index admission mortality as an end point. Utilisation of preoperative chemotherapy was not incorporated into the matrix due to a high proportion of missing data. Additionally, more granular hospital characteristics such as geographical location and academic institution status were not released to ensure that surgeon- and hospital-level data remain anonymous. Our results suggest that a minimally invasive approach was associated with high-quality surgery, which is in contrast with the results of the RAZOR study [37]. This may be related to the fact that such institutions would have adopted a robotic platform, and the variables that we included in our multivariate regression model may not have accounted for other unknown confounding factors. It is worth nothing that the RAZOR study represents a noninferiority study with an oncological outcome primary end point and is not without limitations [38]. PSM was defined as soft tissue PSM and/or CIS, and we acknowledge that this may not entirely reflect quality surgery as ureteral frozen section is not the standard practice and oncological relevance of CIS PSM is debatable. Finally, patient-reported quality of life outcomes were not captured within this dataset.

## 5 Conclusions

We report that there remains a significant association between annual surgeon operating volume and minimally invasive RC, with the attainment of high-quality surgery in patients. Patients treated with high-quality surgery were predicted to have lower index admission mortality. Our results support the role of centralisation of complex oncology as well as the implementation of a high-quality assurance programme to improve the delivery of care for complex oncological surgery such as RC.

  ***Author contributions*:** Wei Shen Tan had full access to all the data in the study and takes responsibility for the integrity of the data and the accuracy of the data analysis.

  *Study concept and design*: Tan, Trinh, Kelly.

*Acquisition of data*: Tan, Fowler.

*Analysis and interpretation of data*: Tan, Leow, Marchese, Sridhar, Hellawell, Mossanen, Teoh, Fowler, Colquhoun, Cresswell, Catto, Trinh, Kelly.

*Drafting of the manuscript*: Tan.

*Critical revision of the manuscript for important intellectual content*: Leow, Colquhoun, Catto, Trinh, Kelly.

*Statistical analysis*: Tan, Marchese.

*Obtaining funding*: None.

*Administrative, technical, or material support*: None.

*Supervision*: Trinh, Kelly.

*Other*: None.

  ***Financial disclosures:*** Wei Shen Tan certifies that all conflicts of interest, including specific financial interests and relationships and affiliations relevant to the subject matter or materials discussed in the manuscript (eg, employment/affiliation, grants or funding, consultancies, honoraria, stock ownership or options, expert testimony, royalties, or patents filed, received, or pending), are the following: None.

  ***Funding/Support and role of the sponsor:*** None.

  ***Acknowledgements*:** Data were provided by the British Association of Urological Surgeons.
