# Supplementary material for: Defining Factors Associated with High-quality Surgery Following Radical Cystectomy: Analysis of the British Association of Urological Surgeons Cystectomy Audit
Source: Eur Urol Open Sci. 2021 Sep 20;33:1–10. doi: 10.1016/j.euros.2021.08.005 (PMC8546928; doi:10.1016/j.euros.2021.08.005)
Supplement: Supplementary file 1 [file mmc1.docx]

Supplementary Table 1: Minimum p value method to determine threshold for annual surgeon and hospital volume to achieve higher rates of high-quality surgery.

A: Surgeon annual volume

| Comparison of surgeon annual cases | Quality surgery (%) | P value |
| --- | --- | --- |
| 1-9  10-92 | 12.2  20.3 | 0.000117721 |
| 1-13  14-92 | 12.8  21.2 | 1.920e-08 |
| 1-16  17-92 | 13.9  21.9 | 7.926e-10 |
| 1-19  20-92 | 14.2  23.0 | 2.118e-13 |
| 1-23  24-92 | 15.7  23.5 | 1.822e-11 |
| 1-26  27-92 | 16.3  24.1 | 4.848e-11 |
| 1-31  32-92 | 17.6  24.2 | 1.837e-07 |
| 1-37  38-92 | 18.7  23.4 | 0.001353711 |
| 1-47  50-92 | 19.1  23.9 | 0.009914721 |

B: Hospital annual volume

| Comparison of hospital annual cases | Quality surgery (%) | P value |
| --- | --- | --- |
| 1-17  18-117 | 12.2  20.3 | 0.000117721 |
| 1-24  25-117 | 13.0  21.0 | 1.700e-07 |
| 1-31  32-117 | 14.9  21.2 | 2.799e-06 |
| 1-38  39-117 | 16.9  21.1 | 0.000621524 |
| 1-47  48-117 | 17.5  21.2 | 0.001673876 |
| 1-52  53-117 | 18.5  20.9 | 0.036424562 |
| 1-61  62-117 | 18.3  22.1 | 0.002166809 |
| 1-66  68-117 | 17.8  24.8 | 9.928e-08 |
| 1-82  84-117 | 19.1  23.4 | 0.009911058 |
